# Fabrication and characterization of unique sustain modified chitosan nanoparticles for biomedical applications

**DOI:** 10.1038/s41598-024-64017-4

**Published:** 2024-06-15

**Authors:** Marwa ElS. Ahmed, Mansoura I. Mohamed, Hanaa Y. Ahmed, Mahmoud M. Elaasser, Nadia G. Kandile

**Affiliations:** 1 Chemistry Department, Faculty of Women for Art, Science and Education, Ain Shams University, Heliopolis, Cairo, 11757 Egypt; 2 Regional Center for Mycology and Biotechnology, Al-Azhar University, Nasr City, Cairo, 11787 Egypt

**Keywords:** Chitosan, Nanoparticle, Antitumor apoptosis HeLa cell, HEp-2 cell line, Biochemistry, Chemistry, Materials science, Nanoscience and technology

## Abstract

Chitosan (CS) is a biopolymer that offers a wide range in biomedical applications due to its biocompatibility, biodegradability, low toxicity and antimicrobial activity. Syringaldehyde (1) is a naturally occurring organic compound characterized by its use in multiple fields such as pharmaceuticals, food, cosmetics, textiles and biological applications. Herein, development of chitosan derivative with physicochemical and anticancer properties via Schiff base formation from the reaction of chitosan with sustainable eco-friendly syringaldehyde yielded the (CS-1) derivative. Moreover, in the presence of polyethylene glycol diglycidyl ether (PEGDGE) or sodium tripolyphosphate (TPP) as crosslinkers gave chitosan derivatives (CS-2) and (CS-3NPs) respectively. The chemical structures of the new chitosan derivatives were confirmed using different tools. (CS-3NPs) nanoparticle showed improvement in crystallinity, and (CS-2) derivative revealed the highest thermal stability compared to virgin chitosan. The cytotoxicity activity of chitosan and its derivatives were evaluated against HeLa (human cervical carcinoma) and HEp-2 (Human Larynx carcinoma) cell lines. The highest cytotoxicity activity was exhibited by (CS-3NPs) compared to virgin chitosan against HeLa cell growth inhibition and apoptosis of 90.38 ± 1.46% and 30.3% respectively and *IC*_*50*_ of 108.01 ± 3.94 µg/ml. From the above results, it can be concluded that chitosan nanoparticle (CS-3NPs) has good therapeutic value as a potential antitumor agent against the HeLa cancer cell line.

## Introduction

Cancer is still the most common cause of morbidity in the world. Cell proliferation is one of the major steps in cancer cells metastasis of a multistep process that includes cell adhesion, invasion, transport through circulatory system and growth in a secondary organ^1^. Cervical cancer is the third incessant cancer type faced by women^2^. In recent years, many efforts on searching for efficient anticancer agents from natural products lead to increasing interest in polysaccharides.

Polysaccharides are obtained from renewable resources, and currently chitosan is intensively studied due to its application in the pharmaceutical, cosmetics, biomedical, biotechnological, agricultural, and food industries^3^. Chitosan is a degradable multifunctional biopolymer obtained by chemical or enzymatic deacetylation of chitin extracted from crustacean exoskeleton, insect cuticle, and fungal mycelium, it contains in its structure a d-glucosamine moiety linked by a β-1,4 glycosidic bond^4–6^.

Chitosan is a cationic polymer that leads to react with the negatively charged molecules. Moreover, the existence of hydroxyl and amine groups in chitosan structure promotes its chemical reactions with heterocyclic compounds^7–12^.

These features make chitosan a valuable and versatile material for biomedical applications. Chitosan has excellent properties such as biocompatibility, biodegradability, bioadhesiveness, non-toxicity, in situ gelling transfection enhancement, bioactive compound/drug release control, permeation enhancement, and mucoadhesion. These properties make chitosan a versatile compound that can be used for a variety of applications^7–9^.

Chemical modification of chitosan is an excellent technique to control the interaction of chitosan with different components. Chitosan reacts with cross-linking agents such as glutaraldehyde, epochlorohydrin^13–15^ and sodium tripolyphosphate (TPP) to enhance its physicochemical properties for different applications^16,17^.

Syringaldehyde, is a naturally occurring unique compound with a variety of bioactive properties, has essential role in biomedicine applications due to the phenolic aldehyde group, which is tested for anti-proliferation activity and other applications such as antioxidants, antifungal or antimicrobial, and anti-tumorigenic agents in pharmaceuticals^18^.

Nanotechnology has increased in many different areas in the last years, including biomedicine, where nanoparticles have been assessed by their potential to be used against different diseases like cancer. Nanoparticles have been shown interesting options in cancer diagnostics and therapeutics^19,20^.

This current study deals with the fabrication of new modified chitosan derivatives and its nanoparticle (CS-1), (CS-2) and (CS-3NPs) via Schiff base formation from the reaction of chitosan with sustainable naturally eco-friendly syringaldehyde (1) in the presence of (PEGDGE) or (TPP) as crosslinkers respectively via microwave reactor to reinforcement chitosan physicochemical properties. The fabricated modified chitosan derivatives were characterized by FTIR, XRD, SEM, EDX, TEM, DLS, TGA, DSC, and Elemental analysis. The biological activity of new chitosan derivatives towards cytotoxicity HeLa cancer (human cervical carcinoma), HEp-2 (Human Larynx Carcinoma) cell lines and mode of action of (CS-3NPs) on apoptotic pathways in HeLa cancer cell line using annexin V-FITC and propidium iodide were investigated.

## Materials and methods

### Materials

Low molecular weight chitosan (CS) (MW 60 KDa, DD 91%) was purchased from (Acros, Belgium), 3,5-dimethoxy-4-hydroxybenzaldehyde (syringaldehyde), acetic acid, poly(ethylene glycol diglycidyl ether) (PEGDGE) and absolute ethanol were imported from (Aldrich Egypt). Sodium tripolyphosphate (TPP) was provided from (Adwic Egypt). All aqueous solutions were prepared using distilled water.

### Methods

#### Preparation of modified chitosan derivatives (CS-1, CS-2 and CS-3NPs)

##### Preparation of chitosan derivative (CS-1)

Chitosan (CS) (0.5 g, 1% w/v) was stirred in dilute acetic acid solution (50 ml, 1% v/v) at room temperature until complete solubility. Syringaldehyde (1) (0.5 g, 0.0027 mol) was dissolved in (20 ml) absolute ethanol then added to (CS) solution. The reaction mixture was stirred (100 rpm) for 30 min until clear solution then irradiated in microwave for (1 min) at 600 W. The reaction mixture was left to cool and the solid formed was washed with absolute ethanol to remove unreacted aldehyde, then the excess of acetic acid neutralized by aqueous NaOH. Finally, the product was washed with distilled water and left to dry in oven at 60 °C to give (CS-1) derivative.

##### Preparation of chitosan derivative (CS-2)

Chitosan (CS) (0.5 g, 1% w/v) was dissolved in aqueous solution of acetic acid (50 ml, 1% v/v) under stirring at room temperature until complete solubility. Syringaldehyde (1) (0.5 g, 0.0027 mol) was dissolved in (20 ml) absolute ethanol, and (PEGDGE) (0.5 ml) added to (CS) solution. The reaction mixture was stirred (100 rpm) for (30 min) until clear solution then irradiated in microwave for (2 min) at 600 W. The solid formed was left to cool, then washed with absolute ethanol to remove unreacted aldehyde, and the excess of acetic acid neutralized by aqueous NaOH, finally, washed with distilled water and left to dry in oven at 60 °C to give (CS-2) derivative.

##### Preparation of chitosan nanoparticle (CS-3NPs)

Chitosan (CS) (0.5 g, 1% w/v) was dissolved in (50 ml acetic acid, 1% v/v) with stirring at room temperature until complete solubility. Syringaldehyde (1) (0.5 g, 0.0027 mol) was dissolved in (20 ml) absolute ethanol and aqueous solution of (TPP) (20 ml, 1% wt/v) was added dropwise with stirring (100 rpm) for (2 h). The reaction mixture was irradiated in microwave for (2 min) at 600 W and left to cool, then washed with absolute ethanol to remove unreacted aldehyde, and the excess of acetic acid neutralized by aqueous NaOH. Finally, the product was washed with distilled water and left to dry in oven at 60 °C to give (CS-3NPs) derivative.

### Characterization of chitosan and its derivatives

#### Determination of the average molecular weight of chitosan

In order to determine the average molecular weight (MW) of chitosan, viscometry measurement was carried out in the Central Lab, National Research Center, Cairo, Egypt using Brookfield viscometer type at 25 °C.

In this method, different concentrations of (CS) were dissolved in mixture of 0.1 M acetic acid and 0.2 M sodium chloride. The viscosity of (CS) solutions and buffer solution were measured triplicated and relative viscosity (η) was calculated. Chitosan (MW) was calculated based on the intrinsic viscosity [η] by Mark–Houwink–Sakurada's empirical equation^21^:

1 $$\left[\eta \right]=K{M}^{a}$$

where k = 1.81 × 10^−3^ and a = 0.93 for the buffer solution at 25 °C.

#### Elemental Analysis

Elemental analysis (C, H, N) was performed using FLASH 2000 CHNS/O analyzer (Thermo Scientific) in the Regional Center for Mycology and Biotechnology Al-Azhar University, Cairo, Egypt. The degree of deacetylation (DD %) for (CS) and degree of substitution (DS) for chitosan derivatives were calculated using the following Eqs. (2) and (3)^10–12,21,22^ respectively.

2 $$ DD\%  = \left( {1 - \frac{{C/N - 5.145}}{{6.861 - 5.145}}} \right) \times$$

3 $$DS\text{\%}=\frac{{(C/N)}_{f} -{(C/N)}_{i}}{n}$$

where the value of 5.145 is related to the completely N-deacetylated chitosan (C_6_H_11_O_4_N) repeat unit and the value of 6.861 is related to the fully N-acetylated chitin (C_8_H_13_O_5_N) repeat unit. (C/N)_f_ and (C/N)_i_ are the ratio of the synthesized polymers and chitosan respectively, also n was the number of carbons introduced into the synthesized polymers.

#### FTIR spectroscopy

Perkin Elmer 200 (FTIR) spectrophotometer instrument was utilized. The samples were grinded with KBr to form a disk and measured in the wavelength range from 4000 to 450 cm^−1^ during 64 scans, with 2 cm^−1^ resolutions.

#### X-ray diffraction (XRD)

X-Ray diffraction (XRD) studies were operated using an X-ray diffractometer (D2 Phaser, Bruker AXS, Germany) operating at 30 kV and 10 mA. The diffraction patterns were also recorded by using Cu-K radiation. The samples were analyzed at 2θ degree range from 1 to 50.

#### Thermo gravimetric analysis (TGA)

Thermogravimetric analysis (TGA) was done at temperatures ranging from 25 to 600 °C. The TGA curves for all the samples were obtained in the inert nitrogen atmosphere with a heating rate of 10 °C/min by using the instrument: SDT Q600 V20.9 Build 20, USA. Capacity of sample: 200 mg (350 mg including sample holder). Balance sensitivity: 0.1 μg. Calorimetric accuracy: ± 2% (based on the metal standards). It features a field-proven horizontal dual beam design with automatic beam growth compensation and the ability to analyze TGA samples simultaneously.

#### Differential scanning calorimetry (DSC)

DSC 131 evo (SETARAM Inc., France) was used to specify the differential scanning calorimeter (DSC) analyses. The instrument was calibrated utilizing mercury, indium, tin, lead, zinc and aluminum as the standards. Nitrogen and helium were used as the purging gases. The tests were programed to include the heating zone from 25 to 500 °C with a heating rate 10 °C/min. The samples were weighed in aluminum crucible 120 μl and introduced to the DSC. The thermogram results were processed using CALISTO Data processing software v.149.

#### Scanning electron microscopy (SEM) and energy-dispersive X-ray spectrometer (EDX)

Microscopic investigations on samples were carried out using a Philips XL30 scanning electron microscope (SEM) equipped with a LaB6 electron gun and a Philips-EDAX/DX4 energy-dispersive X-ray spectrometer (EDX). Images were taken at different magnifications (from 1509 to 30,009), using scanning electron microscope (SEM) in accordance with the clarity of the images. Samples were fixed with carbon glue and metalized by gold vapor deposition to record images.

#### Transmission electron microscopy (TEM) and dynamic light scattering (DLS) evaluations

Shape and size of the nanoparticles were obtained using Transmission Electron Microscope (TEM). The samples were determined by a carbon-coated copper grid. The coated grids were already viewed under a JEM—1200 EX 2, Electron Microscope Jaban Specimens for (TEM) measurements. The particle size and stability of nanoperticles in terms of (DLS) and zeta potential were evaluated using Nano-ZS, Melvern Instruments LTD., (UK).

### In vitro cytotoxicity study

#### Evaluation of antitumor activity

The antitumor activity of chitosan (CS) and modified chitosan derivatives (CS-1), (CS-2), and (CS-3NPs) were tested against two cell lines, namely, the HeLa (human cervical carcinoma), and HEp-2 (Human Larynx Carcinoma), obtained from VACSERA Tissue Culture Unit. The cells were propagated in RPMI-1640 supplemented with 10% heat-inactivated fetal bovine serum, 1% l-glutamine, HEPES buffer, and 50 µg/ml gentamycin. The Cells were treated with compounds ranging from 500 to 3.9 µg/ml and incubated for 24 h at 37 °C after being allowed to adhere for the first 24 h till confluence. After the end of the incubation period, media were aspirated, and the crystal violet solution (1%) was added to each well for at least 30 min. The stain was removed, and the plates were rinsed using tap water until all excess stains were removed. Glacial acetic acid (33%) was added, and then the absorbance of the plates was measured after gently shaking on a Microplate reader (TECAN, Inc.), using a test wavelength of 490 nm^23,24^.

#### Microscopic studies

After the end of the treatment at the tested concentration, the plates were inverted to remove the medium, the wells were washed three times with 300 µl of phosphate buffered saline (pH 7.2), and then the cells were fixed to the plate with 10% formalin for 15 min at room temperature. The fixed cells were stained with 100 µl of 0.25% crystal violet for 20 min. The stain was removed, and the plates were rinsed using deionized water to remove the excess stain and then allowed to dry.

The cellular morphology was observed using an inverted microscope (CKX41; Olympus, Japan) equipped with a digital microscopy camera to capture the images representing the morphological changes compared to control cells. The cytopathic effects (morphological alterations) were microscopically observed at 100×^25^.

#### Apoptosis detection with flow cytometry (ANNEXINV-FITC-propidium iodide staining PI)

In order to investigate the type of cell death induced by samples of (CS), (CS-1), (CS-2), and (CS-3NPs), flow cytometric analysis was done by performing a dot plot assay. HeLa cell line was treated with 125 µg of different compounds for 24 h. Untreated cells (control) were also included in the experiment design. Approximate 1 × 106 cells were harvested, washed with ice-cold PBS twice, and centrifuged for 15 min at 500 × g at 4 °C. The supernatant was discarded, and the cell pellets were resuspended in ice-cold 1× Binding Buffer and then incubated on ice. After that, 1 µl of annexin V-FITC solution and 5 µl of PI solution were added to 100 µl of the cell suspensions. The stained cells were gently mixed and then incubated for 15 min in the dark on ice. After the incubation period, 400 µl of ice-cold 1× binding buffer was added to each tube, with gentle mixing for 5 min, then analyzed by flow cytometry (FACS Caliber (BD FACS Caliber) within 30 min^26^.

### Statistical analysis

The data was encoded and inputted using the statistical software SPSS V.22. The data were evaluated for meeting the assumptions of parametric testing. Continuous variables were analyzed using the Shapiro–Wilk and Kolmogorov–Smirnov tests for normality. Probability and percentile data were standardized for normality using Arcsine Square Root. The data were given as the mean and standard deviation. ANOVA analyses were done for experimental compounds, analysis was run in three replicates at least for each group; post-hoc analysis evaluated using Tukey pairwise comparison; P-values were showed significant at ≤ 0.05, test evaluated using MiniTab V 14. All values are expressed as the mean ± standard error (SE).

## Results and discussion

### Fabrication of new modified chitosan derivatives

**Figure 1:**
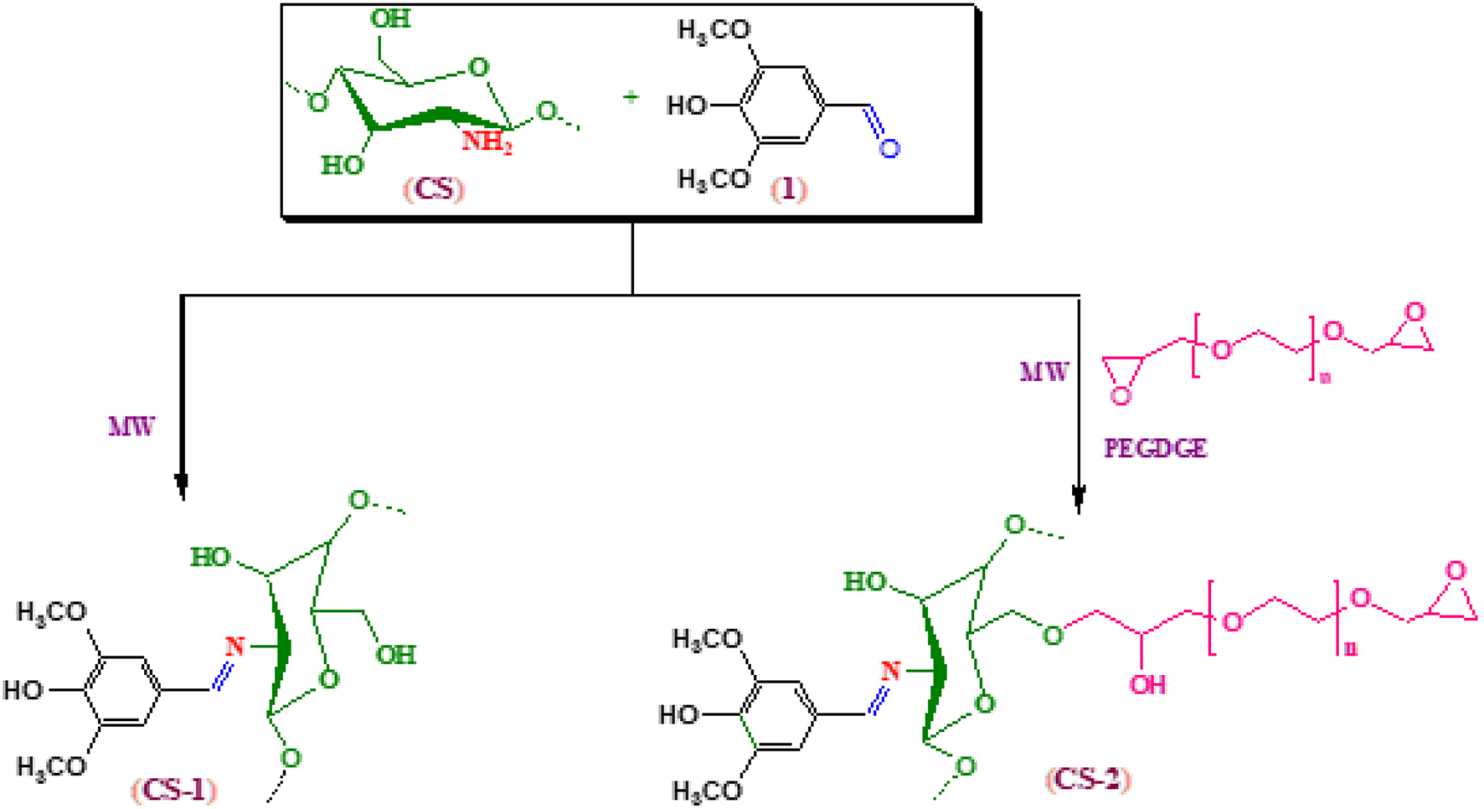
Synthesis of (CS-1), (CS-2) derivatives.

**Figure 2:**
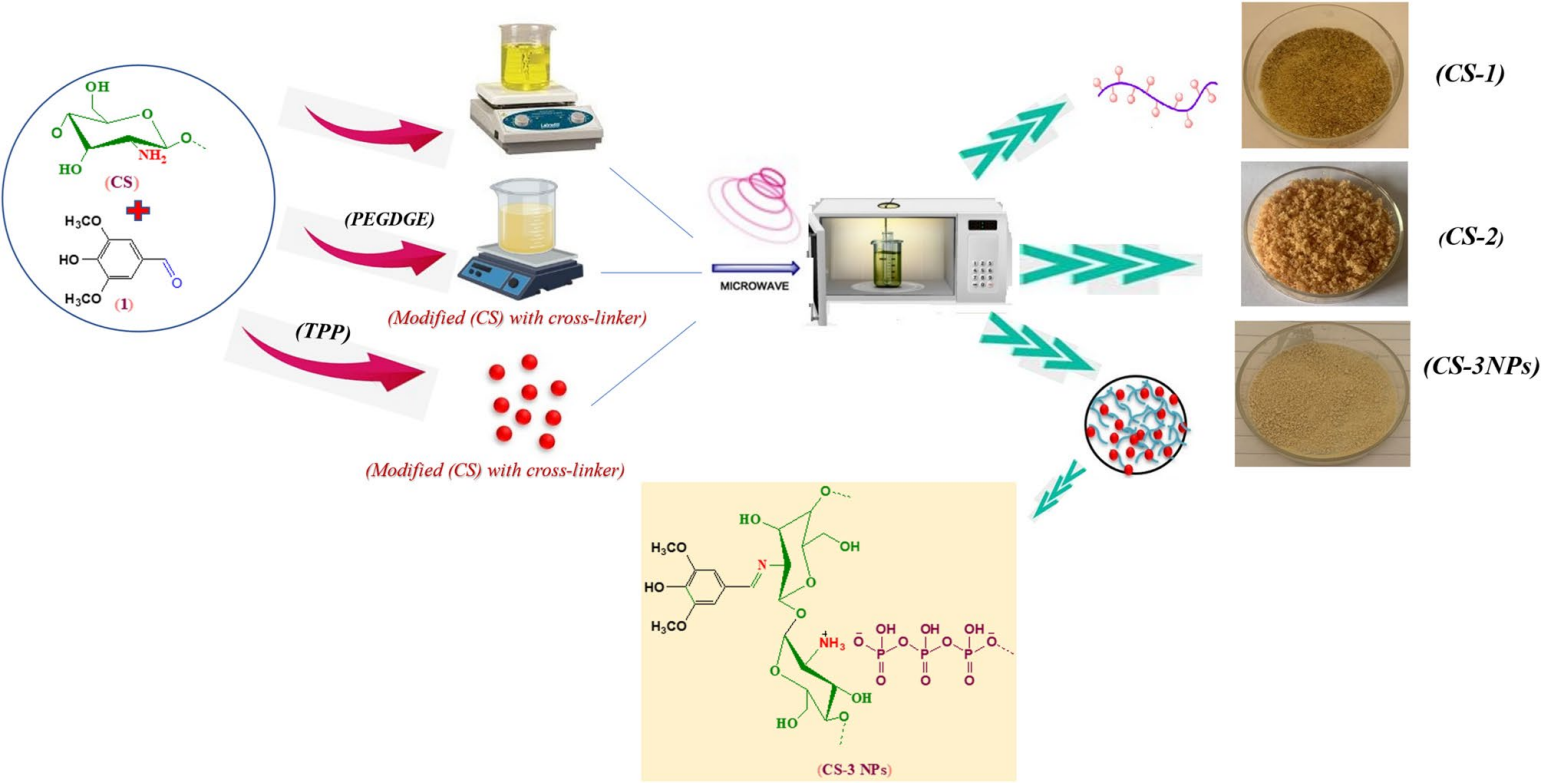
Synthesis of (CS-1), (CS-2) and (CS-3NPs) nanoparticle derivatives.

**Figure 3:**
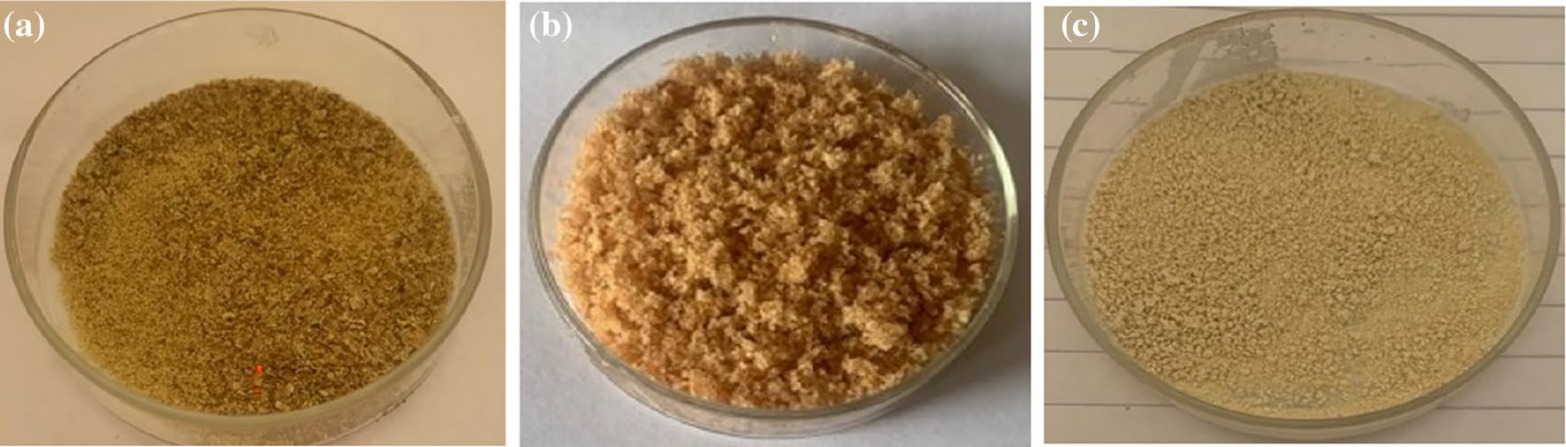
Modified (CS) derivatives (**a**) (CS-1), (**b**) (CS-2), (**c**) (CS-3NPs).

In this study, modified chitosan derivative (CS-1) was fabricated from the reaction of (CS) with syringaldehyde. The mechanism of this reaction was involved nucleophilic attack of free amine group of chitosan backbone with the carbonyl group of aldehyde via Schiff base formation. However, modified chitosan derivative (CS-2) was fabricated via Schiff base formation from the reaction of (CS) with syringaldehyde through the epoxy ring opening of crosslinker (PEGDGE)^10,11^ (Figs. 1, 2, 3).

Also, (CS-3NPs) was prepared through ionic gelation technique between the positively charged amine group of chitosan and negatively charged phosphate group of the crosslinker (TPP)^10,11^ (Figs. 2, 3).

### Characterization of chitosan and chitosan derivatives

#### Viscometry and elemental analysis

The average molecular weight (MW) of chitosan was established by Mark–Houwink–Sakurada's viscometry method and it was approximately 60 KDa. Furthermore, the degree of deacetylation (DD%) and degree of substitution (DS) for (CS) and its derivatives were calculated using elemental analysis represented in Table 1.

**Table 1:** Elemental analysis, (MW), (DD) and (DS) of (CS) and (CS) derivatives.

Sample code	C%	H%	N%	C/N	MW (KDa)	DD%	DS
CS	40.72	7.47	7.68	5.30	60	91	–
CS-1	48.57	5.80	3.41	14.24	–	–	0.9
CS-2	55.29	7.39	2.78	19.88	–	–	0.8
CS-3NPs	35.26	5.28	3.79	9.30	–	–	0.4

From elemental analysis, the degree of deacetylation for (CS) used in this study was 91%. However, from data in Table 1, it was observed that the degree of substitution (DS) of chitosan derivatives (CS-1) and (CS-2) was higher than (CS-3NPs) due to the direct interaction between free amine groups of chitosan backbone with the carbonyl group of syringaldehyde^10,11^. Additionally, (CS-3NPs) showed the lowest (DS) may be attributed to the cross-linker (TPP) which competed with the carbonyl group of syringaldehyde in the reaction with amine group in the backbone of (CS).

#### FTIR spectroscopy

**Figure 4:**
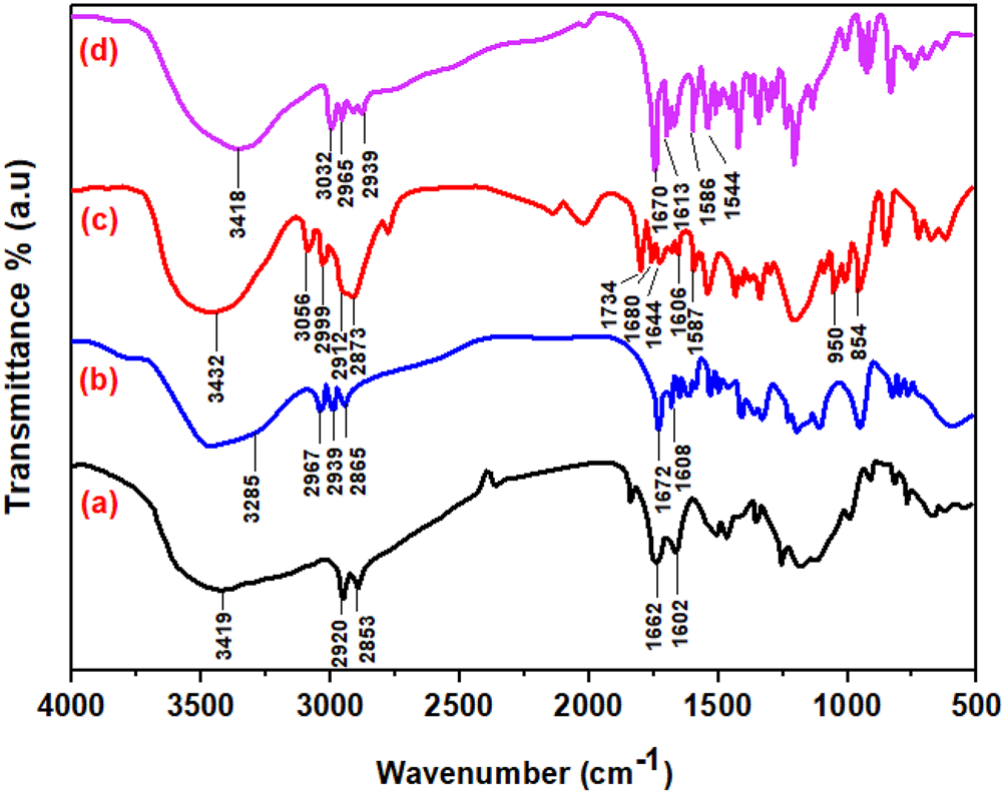
FTIR of (**a**) (CS), (**b**) (CS-1), (**c**) (CS-2) and (**d**) (CS-3NPs).

Figure 4 showed FTIR spectra for (CS), (CS-1), (CS-2) and (CS-3NPs), respectively. The FTIR spectrum of chitosan (CS) exhibited major peaks at 3419 cm^−1^ for stretching vibrations of OH and NH (NH_2_), and at 2920 cm^−1^ referred to C–H aliphatic stretching vibration and peaks at 1662 and 1602 cm^−1^ due to C=O of acetyl and NH groups bending vibration, respectively^10,27^.

Chitosan derivative (CS-1) was characterized by absorption band of the interfering between stretching vibrations OH of syringaldehyde and NH (NH_2_) for chitosan at 3285 cm^−1^. New band appeared at 1672 cm^−1^ for C=N group which confirmed the formation of Schiff base^7,28^.

On the other hand, the spectrum of chitosan derivative (CS-2) showed absorption band intensity at 2912 and 2873 cm^−1^ of C–H chain aliphatic stretching vibration of (PEGDGE), the peak at 950 cm^−1^ corresponding to ether linkage^10,29,30^ and band at 854 cm^−1^ recognized to the epoxy ring^10,31^.

Furthermore, (CS-3NPs) revealed a broad band at 3418 cm^−1^ for OH stretching vibrations of syringaldehyde and NH (NH_2_) of chitosan, this showed enhancement intermolecular hydrogen bonding due to crosslinking with (TPP)^16,17,32^. Another band at 1670 cm^−1^ of C=N group, two bands appeared at 1544 cm^−1^ and 1140 cm^−1^ of (P=O)^33^ and two peaks at 1035 and 869 cm^−1^ (PO_3_^–2^)^34,35^ due to the amine group cross-linked with (TPP).

#### X-ray dffraction (XRD)

**Figure 5:**
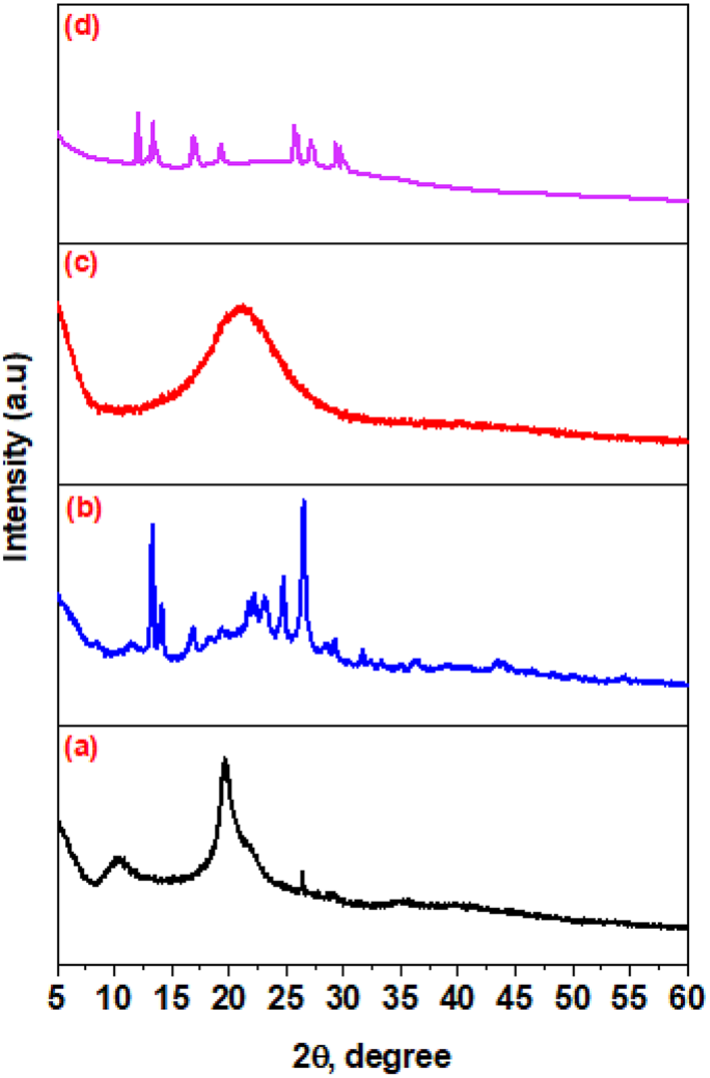
XRD diffraction pattern of (**a**) (CS), (**b**) (CS-1), (**c**) (CS-2) and (**d**) (CS-3NPs).

XRD analyses were performed to determine the physical variation of crystal structures and phase composition of (CS) and its derivatives (CS-1), (CS-2) and (CS-3NPs) (Fig. 5). It can be observed that, the crystal structure of chitosan (CS) is attributed to a semi-crystalline behaviour due to its internal arrangement of monomer units and hydrogen bonding^36^. XRD for virgin chitosan was exhibited two peaks at 2θ = 10.46° and 19.63°^37^.

Chitosan derivative (CS-1) revealed three new shifted peaks at 2θ = 13.30°, 14.11° and 16.84° and two sharp peaks appeared at 23.13° and 26.52° which confirmed the modification of chitosan. However, (XRD) of derivative (CS-2) showed crystalline forms of chitosan peaks at 2θ = 24.04° by increasing cross-linking degree after addition of (PEGDGE), the crystal structure form between crystalline and amorphous phases led to destroy the strong hydrogen bonding in chitosan crystals due to the substitution of hydroxyl and amine groups^38^.

On the other hand, (XRD) of (CS-3NPs) showed four new peaks at 2θ = 13.14°, 14.01°, 16.77° and 18.43° with decrease in intensity, due to the prescence of tripolyphosphate which forming ionic crosslinking between the amine groups of chitosan and the phosphate groups of TPP. This crosslinking can induce a more ordered structure, enhancing the crystallinity of the composite^39–41^.

This indicated that (CS-3NPs) showed improvement in crystallinity compared to virgin (CS). Thus chemical modification of chitosan enhanced the crystallinity of new chitosan derivatives^39–41^.

#### Scanning electron microscopy (SEM)

**Figure 6:**
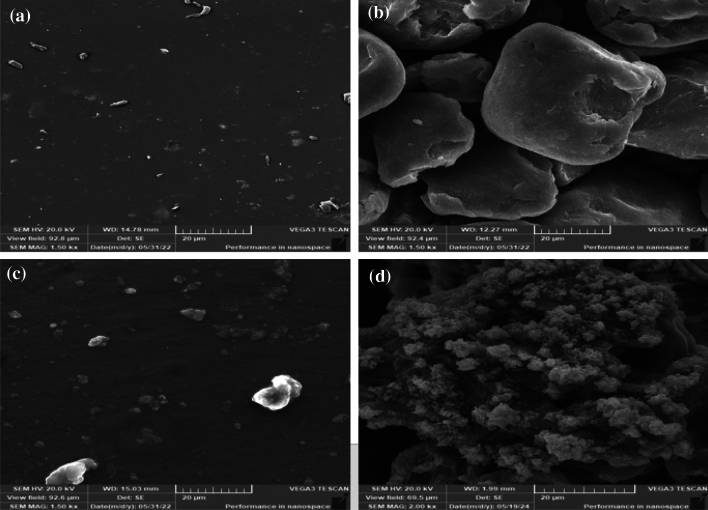
SEM of (**a**) (CS), (**b**) (CS-1), (**c**) (CS-2) and (**d**) (CS-3NPs).

Scanning electron microscopy (SEM) was one of the high-resolution electron microscopy techniques which used to study and characterize the surface morphology of chitosan and new chitosan derivatives. Scanning Electron Microscopy (SEM) photomicrographs of (CS), (CS-1), (CS-2) and (CS-3NPs) were shown in Fig. 6a–d.

Chitosan showed smooth and flat morphology of (CS)^10,11,39^ (Fig. 6a), while Fig. 6b revealed that (CS-1) had a rough surface. Also, the surface morphology of (CS-2) showed irregular structure in Fig. 6c. The (SEM) images of (CS-1) and (CS-2) showed a different morphology compared to virgin (CS) and these different results attributed to the crosslinking degree^10,11,42^. The surface morphology of modified chitosan nanoparticle (CS-3NPs) Fig. 6d appeared rough with a homogeneous distribution of the in-situ generated (TPP) into the surface of modified chitosan. The electrostatic interaction of tripolyphosphoric group of (TPP) and amine group of chitosan resulted in suitable uniform distribution of the generated (TPP)^10,11,41^.

#### Energy dispersive X-ray spectrometer (EDX)

**Figure 7:**
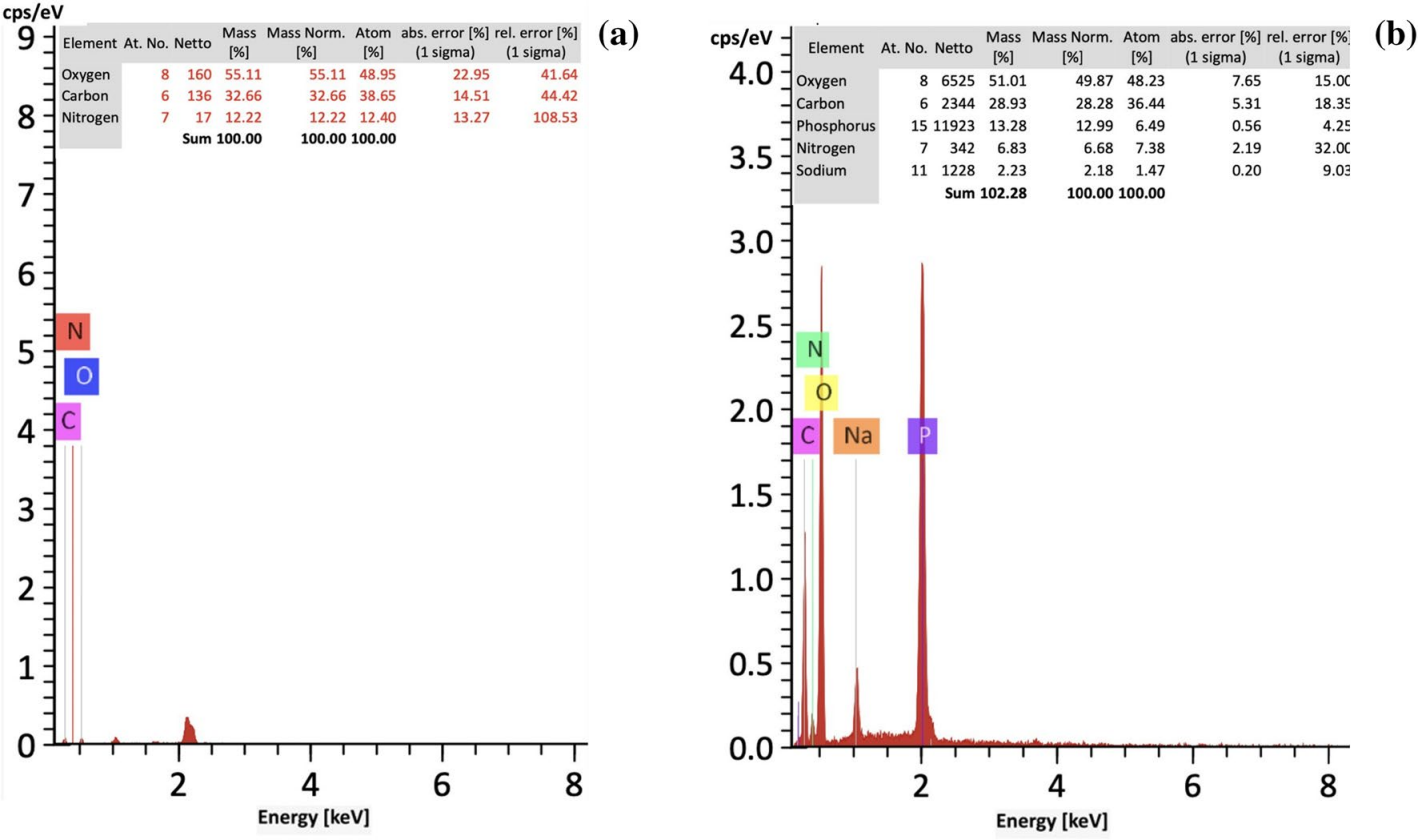
EDX of (**a**) (CS) and (**b**) (CS-3NPs).

EDX technique was used to confirm chitosan (CS) and in-situ reaction of (TPP) with modified chitosan nanoparticle (CS-3NPs) as shown in Fig. 7a,b. From (EDX) charts, it observed that oxygen, carbon, nitrogen, sodium and phosphorus elements were identified. (CS-3NPs) nanoparticle included 36.44% carbon, 48.23% oxygen, 7.38% nitrogen, 1.47% sodium and 6.49% phosphorus compared with virgin (CS) which included 38.65% carbon, 48.95% oxygen and 12.40% nitrogen. The peak corresponding to sodium and phosphorus (indicated arrow) proved the successfully formed and homogeneous dispersion within chitosan matrix.

#### Transmission electron microscopy (TEM)

**Figure 8:**
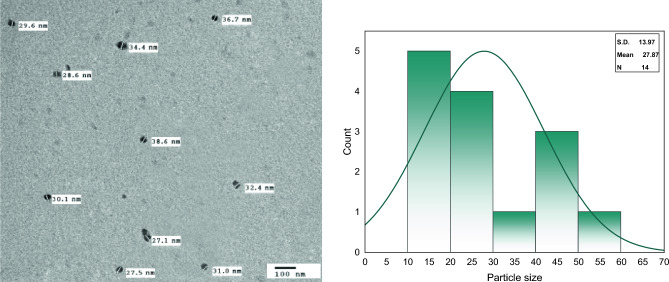
TEM of modified chitosan nanoparticle (CS-3NPs) at 100 nm and particle size distribution diagram.

Transmission electron micrographs (TEM) studied the particle size distribution and spherical morphology of (CS-3NPs) at 100 nm (Fig. 8). (CS-3NPs) revealed a particle size diameter in the range of 27.1–38.6 nm, respectively. These results confirmed the formation of ionic gelation bond between tripolyphosphoric group of (TPP) and amine group of chitosan which caused the change in inter and intramolecular action of chitosan due to the regular and spherical shape of chitosan derivative^10,11,41,43^.

#### Dynamic light scattering (DLS) evaluation

**Figure 9:**
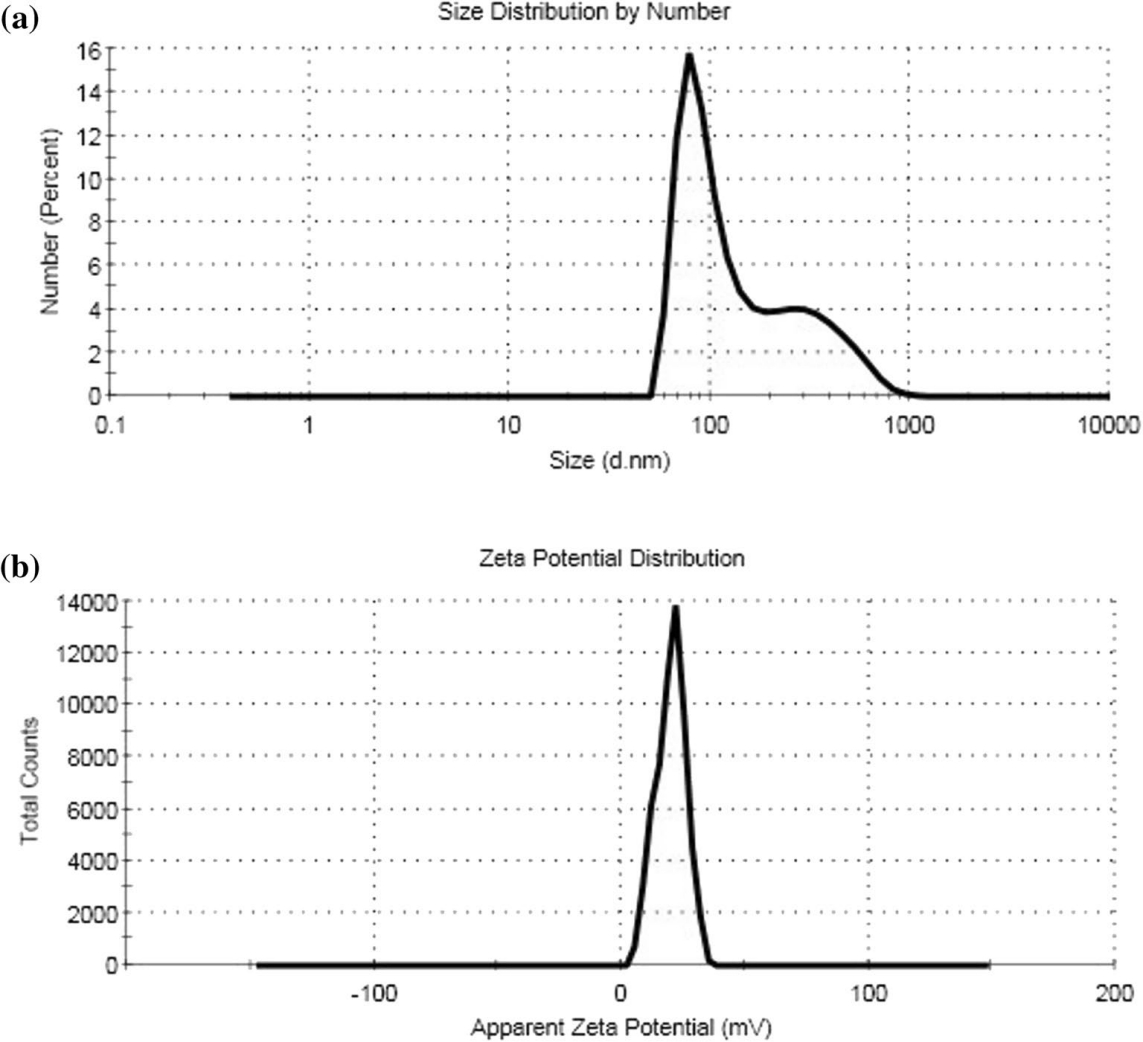
(**a**) Particle size analyzer and (**b**) zeta potential of modified chitosan nanoparticle (CS-3NPs).

Data of (DLS) showed the average particle size at 100 nm (70%) (Fig. 9a). It can be concluded that, the particle size obtained from (DLS) is greater than that of (TEM) analysis Fig. 8.

(DLS) is mainly focused on sample examination in a dispersed form (hydrodynamic diameter). Thus, the hydrodynamic size of the particles is greater, which, increase the particle size. However, the two measurements (TEM and DLS) affirmed the preparation of particles in small size (less than 100 nm) which is suitable for various applications, particularly, biomedical application. In addition, the zeta potential, which represents the surface charge of nanoparticles, greatly influences the stability of the sample. Nanoparticles with higher positive or negative zeta potential values produce greater repulsive forces. The repulsion between nanoparticles with high and similar charge prevents nanoparticles from aggregation. This repulsive force facilitates redispersion and enhances the overall stability of the product. The value ± 20 mV is very desirable for nanoparticles to be stabilized through a combination of electrostatic and steric forces^44^. Zeta potential value for (CS-3NPs) recorded 20 ± 5.98 mV Fig. 9b which gives us a prediction about the stability of the formed nanoparticles.

#### Thermal stability

##### Thermo gravimetric analysis (TGA)

**Figure 10:**
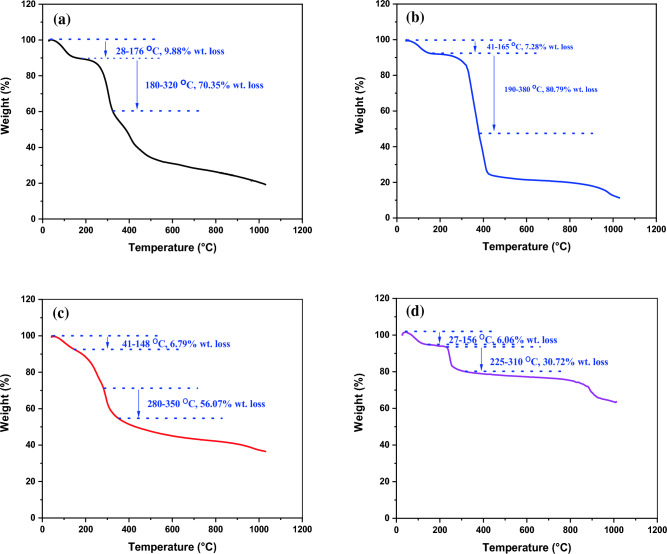
TGA of (**a**) (CS), (**b**) (CS-1), (**c**) (CS-2) and (**d**) (CS-3NPs).

The thermal properties of the virgin chitosan (CS), (CS-1), (CS-2) and (CS-3NPs) are shown in Fig. 10a–d and Table 2. Chitosan (CS) showed two different decomposition stages the first occurring in the range of 28.97–176.18 °C, which attributed to free water molecules at a weight loss of about 9.88%^45^. Water molecules interact with two polar groups, which are weaker than those containing hydroxyl groups, so water molecules attached to amine groups can be more easily removed at lower temperature than molecules attached to hydroxyl groups^46^. TGA of the second stage, weight loss of 70.35% at 180–320 °C attributed to the degradation of glycoside bond of chitosan^47,48^.

**Table 2:** Thermal stability TGA and DSC of (CS), its derivatives (CS-1), (CS-2) and (CS-3NPs).

Sample code	Temperature	Weight loss (%)	Temperature	Weight loss (%)	Temperature	Weight loss (%)	*Tg*	*Tc*
CS	65.4	9.88	–	–	250	70.35	99.69	308.79
CS-1	82	7.28	–	–	285	80.79	105.77	325.89
CS-2	95.26	6.79	–	–	315	56.07	111.25	295.48
CS-3NPs	89	6.06	–	–	295	30.72	83.30	243.99

Thermogravimetric Analysis (TGA) of (CS-1) showed two distinct stages, the first stage showed weight loss of 7.28% range of 41.77–165.11 °C corresponding to the evaporation of free water. The second weight loss was about 80.79% ranged from 190 to 380 °C due to the thermal decomposition of chitosan and chitosan derivative backbone. Thermal analysis of derivative (CS-2) revealed two weight losses: The first weight loss due to depolymerization of chitosan backbone, 6.79% at 41.86–148.67 °C, and the second weight loss of 56.07% at 280–350 °C, due to further destruction of the crosslinking (PEGDGE) as well as the side chain of syringaldehyde^10,11^.

While the (TGA) results of (CS-3NPs) showed two weight loss, the first weight loss 6.06% at 26.95–156.62 °C due to water removal, the second weight loss 30.72% at 225–310 °C due to degradation of the backbone of chitosan and chitosan derivative^10,11,41^.

The (TGA) results demonstrated that the thermal stability of (CS) after modification with crosslinker^10,11,41^. Form gravimetric analysis (TGA) result of (CS-2) showed the highest thermal stability, may be attributed to cross-linking with (PEGDGE).

##### Differential scanning calorimeter (DSC)

**Figure 11:**
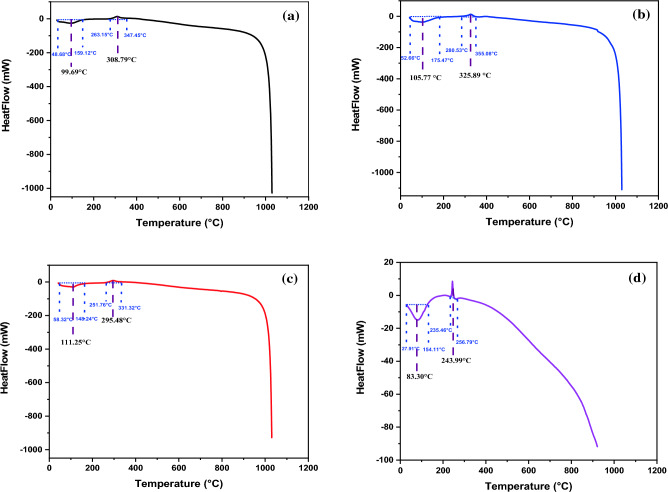
DSC of (**a**) (CS), (**b**) (CS-1), (**c**) (CS-2) and (**d**) (CS-3NPs).

Differential scanning calorimetry (DSC) thermograms was done to investigate the glass transition temperature (Tg) of chitosan and its derivatives. Figure 11a–d and Table 2 illustrated (DSC) thermograms of (CS), (CS-1), (CS-2) and (CS-3NPs). DSC curve of (CS) showed an endothermic glass transition temperature (Tg) at 99.69 °C this may be associated with the removed of moisture^49^. Another exothermic peak revealed to crystalline temperature (Tc) exhibited at 308.79 °C corresponding to the thermal decomposition of pyranose ring^50^. DSC curve of (CS-1) revealed an endothermic (Tg) at 105.77 °C and exothermic peak (Tc) at 325.89 °C. However, (DSC) curve of (CS-2) showed an endothermic glass transition temperature (Tg) at 111.25 °C, which increased than (CS-1), due to crosslinking with long aliphatic carbon chain of (PEGDGE) and increased the segmental motion of the chains^51,52^, and another exothermic peak crystalline temperature (Tc) at 295.48 °C.

On the other hand, (DSC) curve of (CS-3NPs) showed an endothermic glass transition temperature (Tg) at 83.30 °C and exothermic peak of crystalline temperature (Tc) at 243.99 °C. DSC analysis proved that (Tg) of (CS-2) represented higher in (Tg) due to using crosslinking (PEGDGE) restricted the movement of molecular chains^53^.

### Evaluation of antitumor activity

Cancer is still one of the most fatal diseases in the world. There is an urgent need for new medications with unique modes of action; hence much research has been performed for new anticancer treatments derived from natural sources, including plants, microorganisms, and marine species^54^. There are different types of cancer, such as human cervical cancer and HEp-2 (Human Larynx carcinoma cells)^55^. Many efforts have been made to identify new anticancer candidates. Biopolymers (CS) as carrier systems for pharmaceuticals and mucoadhesive formulations have been employed to promote sustainability in the pharmaceutical area^56^ and because of their low toxicity, biodegradability, and colloidal stability in aqueous solutions^57,58^.

**Figure 12:**
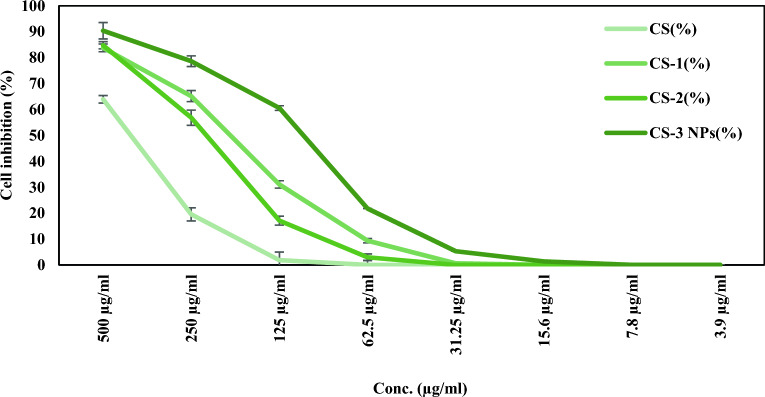
The antitumor activity of (CS), (CS-1), (CS-2), and (CS-3NPs) against HeLa cells. Data were demonstrated as a means of standard deviation (± SD).

**Figure 13:**
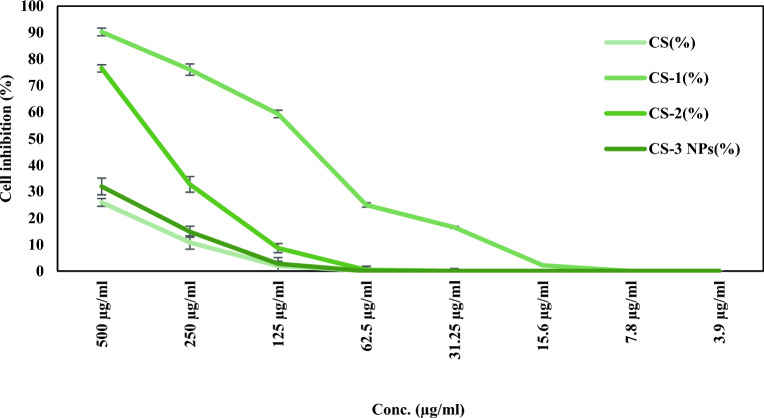
The antitumor activity of (CS), (CS-1), (CS-2), and (CS-3NPs) against Hep-2 Data were demonstrated as a means of standard deviation (± SD).

In continuation of our previous work^11^, the current study revealed the antitumor activity of (CS), (CS-1), (CS-2), and (CS-3NPs) against two types of cancer cells, the HeLa (Human Cervical Carcinoma) and HEp-2 (Human Larynx carcinoma), using different concentrations ranging from 3.9 to 500 µg/ml. It was observed from the result in Fig. 12 that the cell viability reduced as sample concentrations increased on all tested compounds. Also, the antitumor activity of chitosan was improved after modification with crosslinking agents (PEGDGE) and (TPP). (CS-3NPs) represented the higher activity with the highest cell growth inhibition of 90.38 ± 1.46% compared to the other modified chitosan derivatives at the 500 µg/ml concentration against the Hela cell line. In the case of HEp-2 cell line, the (CS-1) derivative possessed higher cell inhibition in comparison with (CS), (CS-2,) and (CS-3 NPs) Fig. 13. The *IC*_*50*_ values, which measure a compound's effectiveness as an antitumor agent, were used to compare the antitumor activity of the investigated compounds Table 3. the findings demonstrated that significant difference (p ≤ 0.05) in which (CS-3NPs) had the lowest *IC*_*50*_ values, approximately 108.01 ± 3.94 µg/ml, followed by (CS-1), (CS-2), and (CS) at approximately 194.44 ± 6.58, 228.51 ± 3.97 and 424.89 ± 12.37 µg/ml, respectively against HeLa cells, compared to HEp-2 cells in which the *IC*_50_ values were recorded about 108.05 ± 2.31, 348.65 ± 4.11, ≥ 500, and ≥ 500 µg/ml, respectively, against (CS-1), (CS-2), (CS), and (CS-3NPs).

**Table 3:** *IC*_50_ values of (CS), (CS-1), (CS-2), and (CS-3NPs) against HeLa cells and HEp-2 cells.

Sample code	*IC*_50_ (µg/ml)	
	Hela cell lines	HEp-2 cell lines
CS	424.89 ± 12.37^a^	≥ 500^b^
CS-1	194.44 ± 6.58^c^	108.05 ± 2.31^d^
CS-2	228.51 ± 3.97^b^	348.65 ± 4.11^c^
CS-3NPs	108.01 ± 3.94^d^	≥ 500^a^

All values were expressed as mean ± standard deviation (n = 3). Values with different superscript letters in a column are significantly different at p ≤ 0.05.

From the above results, it was observed that the HeLa cells were more affected by all tested compounds. However, low cytotoxic effects were observed against (CS), and (CS-3NPs) at the same concentration against HEp-2 cells. So, the current study results indicate that the tested compounds inhibited the growth and the metabolic activities of HeLa and HEp-2 cells in a concentration-dependent manner. The degree of growth inhibition was cell-specific.

### Morphological changes

**Figure 14:**
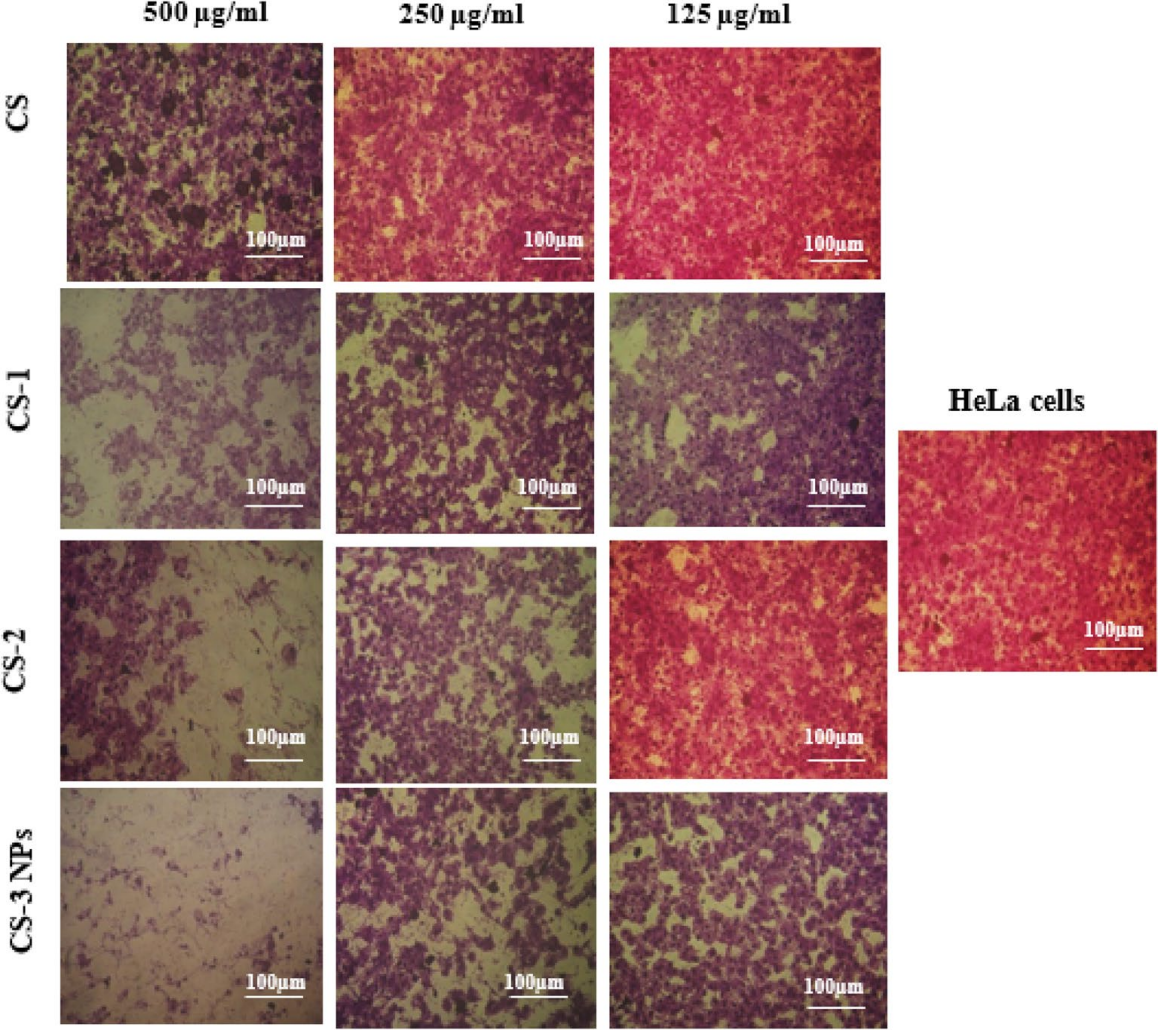
Morphological changes of HeLa cells for (CS), (CS-1), (CS-2), and (CS-3NPs).

**Figure 15:**
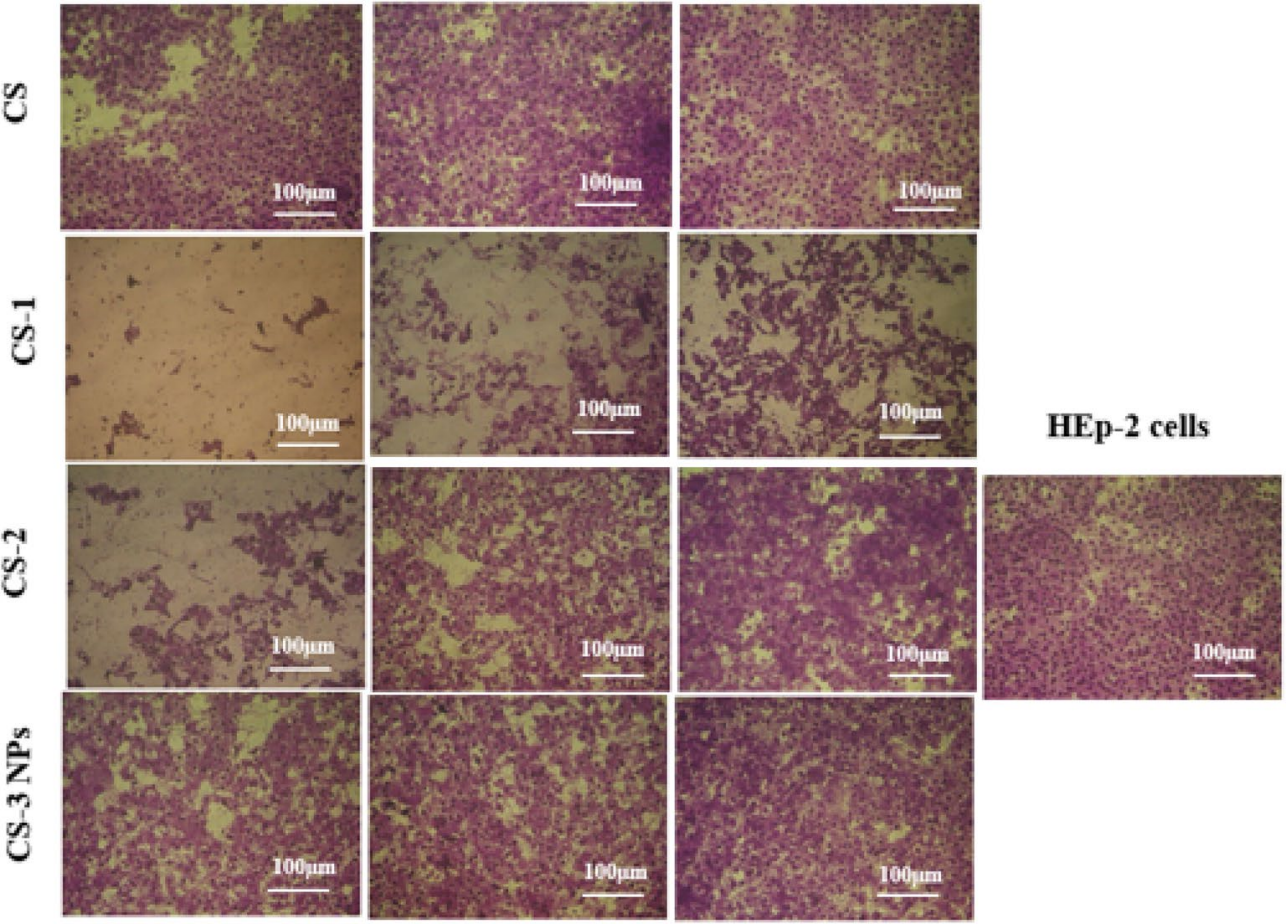
Morphological changes of HEp-2 cells for (CS), (CS-1), (CS-2), and (CS-3NPs).

The results of morphological changes of the images taken by the inverted microscope of HeLa and HEp-2 cells for (CS), (CS-1), (CS-2) and (CS-3NPs) at concentrations from 500 to 125 µg/ml, which confirmed the increase in the percent of live cells with an increase in dilution for each treatment from 500 to 125 µg/ml, in which higher morphological changes were observed at higher concentrations for (CS-3NPs) followed by (CS), (CS-1), and (CS-2) as compared to the untreated control^59^. In contrast, the higher changes on HEp-2 cells were detected for (CS-1) followed by (CS-2). Also, low cytotoxic effects were observed after treatment with (CS), and (CS-3NPs) (Figs. 14, 15).

### Induction of apoptosis by modified chitosan derivatives in HeLa cells

Among the two tested cell lines (HeLa cells and HEp-2), the HeLa cell line was the most affected against all tested compounds, so it was selected to investigate the mode of action by quantifying the apoptosis percentage caused by tested compounds.

Apoptosis, or programmed cell death, is a natural mechanism that removes unnecessary or damaged cells with potentially dangerous mutations during embryonic development and tissue remodeling^60^. Deregulation of apoptosis can break the delicate balance between cell proliferation and cell death, leading to illnesses such as cancer^61^. Chitosan (CS) is a promising medication delivery biopolymer^62,63^ with strong functional groups, such as amine groups, that can react with various compounds, resulting in a diverse biodegradable polymer suitable for biomedical applications^63^. It progressively degrades into harmless byproducts absorbed by the human body^64^. It is given that (CS) can interact with the tumor microenvironment to either suppress or induce apoptosis in cells^65,66^. Moreover, (CS) is a mucoadhesive cationic polymer that may have a greater affinity for negatively charged cell membranes, and can enhance cellular absorption, lengthen retention time inside the body, and consequently increase drug bioavailability^65,67^.

Chitosan (CS), modified chitosan derivatives (CS-1), (CS-2), and (CS-3NPs) were tested at concentrations of 125 µg to evaluate its apoptotic effects on the HeLa cell line by flow cytometry.

**Figure 16:**
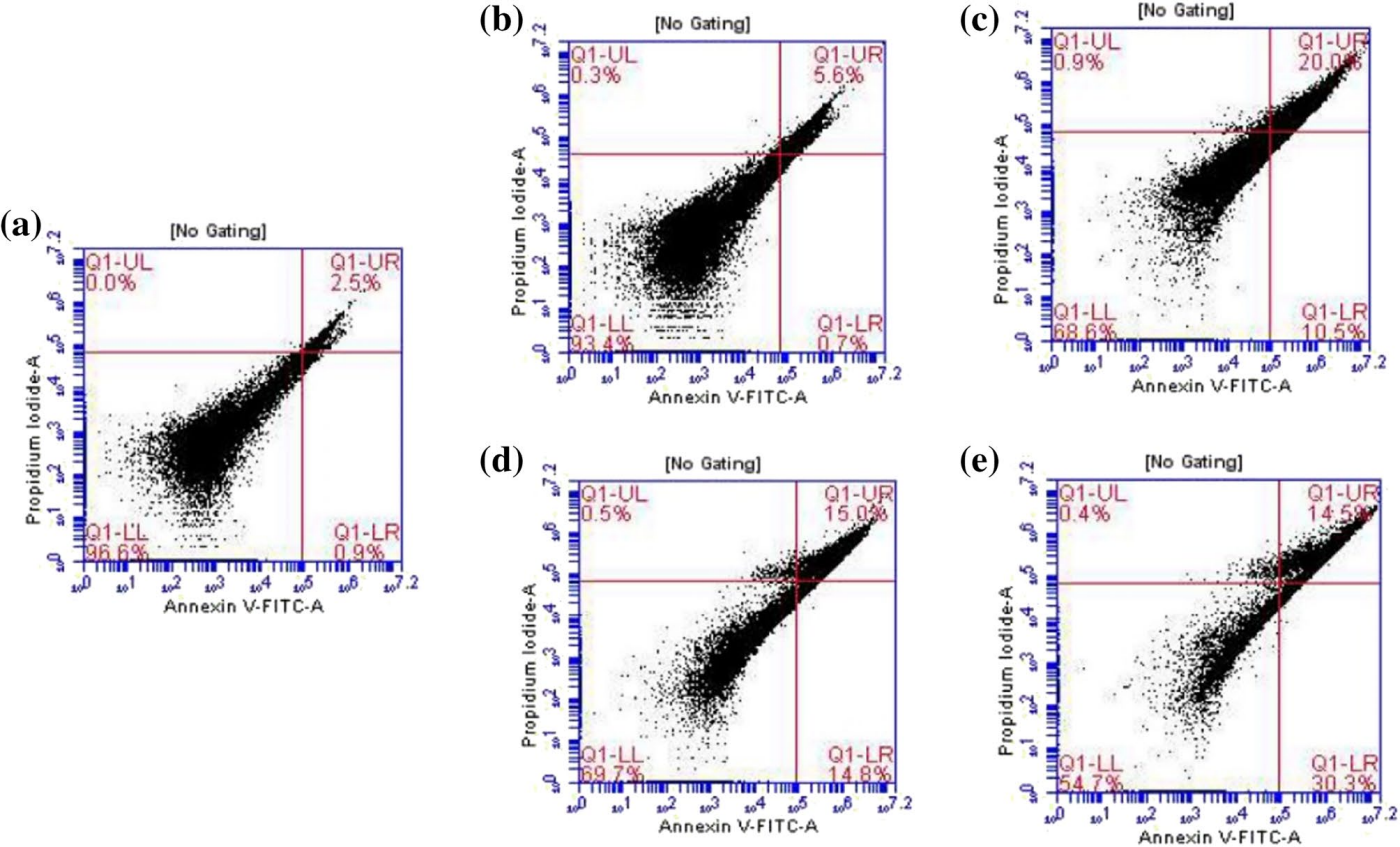
Flow cytometric analysis of treated HeLa cells with 125 µg of different derivatives. (**a**) Control, (**b**) Chitosan (CS), (**c**) (CS-1), (**d**) (CS-2), and (**e**) (CS-3NPs). The lower left (LL) quadrant (Q1) (annexin V−/PI−) is regarded as the population of live cells, the lower right quadrant (Q2) (LR) (annexin V+/PI−) is considered as the cell population at the early apoptotic stage, the upper right (UR) quadrant (Q3) (annexin V+/PI+) represents the cell population at the late apoptotic stage, and upper left (UL) quadrant (Q4) (annexin V−/PI+) is considered as necrotic cell population.

Figure 16a–e illustrates the dot plots of Annexin-V/propidium iodide staining. The lower left (LL) quadrant (annexin V−/PI−) is regarded as the population of live cells, the lower right quadrant (LR) (annexin V+/PI−) is considered as the cell population at the early apoptotic stage. The upper right (UR) quadrant (annexin V+/PI+) represents the cell population at the late apoptotic stage, and the upper left (UL) quadrant (annexin V−/PI+) is considered as necrotic cell population.

Flow cytometric analysis revealed that HeLa cells, after 24 h of treatment with 125 µg of chitosan (CS), about 0.7% of cells were in LR quadrant (early apoptotic stage), and 5.6% of the HeLa cell line were in UR quadrant (late apoptotic stage) (Fig. 16b). While, after treatment with modified chitosan (CS-1) at the same concentration, about 10.5% of cells were in LR quadrant (early apoptotic stage) and 20% of cells were in UR quadrant (late apoptotic stage) (Fig. 16c).

However, modified chitosan (CS-2), about 14.8% of cells were in the LR quadrant (early apoptotic stage), and 15% of cells were in the UR quadrant (late apoptotic stage) (Fig. 16d). In contrast, about 30.3% of cells were in LR quadrant (early apoptotic stage), and 14.59% of cells in UR quadrant (late apoptotic stage) after treatment with modified chitosan (CS-3NPs) (Fig. 16e). As compared with untreated control the majority of cells viable and appeared in the lower left quadrant because they bound neither the Annexin V nor the PI dyes (Fig. 16a).

From the above finding, it has been confirmed that modified chitosan (CS-3NPs) induced cell death via apoptosis with significant (p ≤ 0.05) higher percent than other derivatives. Chemical modification of chitosan in nanoformulation could overcome multidrug resistance and improve cancer treatment efficacy, especially toward resistant cells^68,69^. Our results agree with reported by Özdamar^70^ in which (CS) nanoparticles were cytotoxic and induced apoptosis in HeLa cells. The most important properties of (CS) nanoparticles could provide a high resemblance used for negatively charged biological membranes and targeting specific sites in vivo^71^.

## Conclusion

In this study, modified chitosan derivatives (CS-1), (CS-2) and (CS-3NPs) were fabricated and characterized. The highest crystallinity and thermal stability were exhibited by (CS-3NPs) and (CS-2) respectively. Moreover, (CS-3NPs) revealed particle size distribution lower than 100 nm in (TEM) images and (DLS) average particle size at 100 nm. However, Zeta potential value for (CS-3NPs) recorded 20 ± 5.98 mV. Additionally, antitumor activity of modified chitosan nanoparticle (CS-3NPs) showed higher cell growth inhibition compared to virgin (CS) and (CS) derivatives against HeLa cancer cell line.

Based on the results of the in-vitro antitumor evaluation, it was observed that (CS-3NPs) nanoparticle represented the highest cell growth inhibition of 90.38 ± 1.46% with the lowest IC50 value at 108.01 ± 3.94 µg/ml against HeLa cancer cell line. While *IC50* (CS-1), (CS-2) derivatives and (CS) 194.44 ± 6.58, 228.51 ± 3.97 and 424.89 ± 12.37 µg/ml respectively. Furthermore, mode of action showed that modified chitosan nanoparticle (CS-3NPs) exhibits anti-apoptotic activity, had a potency of treating HeLa cell line in significant way that can be considered as a promising new pathway in cancer therapeutics.

## Data Availability

All data generated or analysed during this study are included in this published article.

## References

[1] Abedian Z (2019). Anticancer properties of chitosan against osteosarcoma, breast cancer and cervical cancer cell lines. Caspian J. Intern. Med..

[2] Ashique S, Upadhyay A, Kumar N, Chauhan S, Mishra N (2022). Metabolic syndromes responsible for cervical cancer and advancement of nanocarriers for efficient targeted drug delivery-A review. Adv. Cancer Biol. Metastasis.

[3] Vitali L, Vieira IC, Spinelli A (2011). Sensor-containing microspheres of chitosan crosslinked with 8-hydroxyquinoline-5-sulphonic acid for determination of Cu (II) in instant coffee. Food Chem..

[4] Kandile NG, Zaky HT, Mohamed MI, Nasr AS, Ali YG (2018). Extraction and characterization of chitosan from shrimp shells. Open J. Org. Polym. Mater..

[5] Zhang M (2022). Application of chitosan and its derivative polymers in clinical medicine and agriculture. Polymers.

[6] Bandara S, Du H, Carson L, Bradford D, Kommalapati R (2020). Agricultural and biomedical applications of chitosan-based nanomaterials. Nanomaterials.

[7] Hamed AA (2022). Chitosan Schiff bases-based polyelectrolyte complexes with graphene quantum dots and their prospective biomedical applications. Int. J. Biol. Macromol..

[8] Deshmukh AR (2021). Biodegradable films based on chitosan and defatted Chlorella biomass: Functional and physical characterization. Food Chem..

[9] Deshmukh AR, Kim BS (2019). Chitosan-vitamin C nanoparticles. KSBB J..

[10] Kandile NG, Mohamed HM (2021). New chitosan derivatives inspired on heterocyclic anhydride of potential bioactive for medical applications. Int. J. Biol. Macromol..

[11] Ali M (2023). New potential anti-SARS-CoV-2 and anti-cancer therapies of chitosan derivatives and its nanoparticles: Preparation and characterization. Arab. J. Chem..

[12] Kandile NG, Mohamed MI, Zaky HT, Nasr AS, Ali YG (2022). Quinoline anhydride derivatives cross-linked chitosan hydrogels for potential use in biomedical and metal ions adsorption. Polym. Bull..

[13] Bratskaya SY (2012). N-(2-(2-pyridyl) ethyl) chitosan: Synthesis, characterization and sorption properties. Carbohydr. Polym..

[14] Bratskaya SY (2013). Synthesis and properties of isomeric pyridyl-containing chitosan derivatives. Int. J. Biol. Macromol..

[15] Cittan M, Tirtom VN, Dinçer A, Çelik A (2014). Epichlorohydrin crosslinked chitosan–clay composite beads for on-line preconcentration and determination of chromium (III) by flow injection flame atomic absorption spectrometry. Anal. Methods.

[16] Chang Y-L, Liu TC, Tsai M-L (2014). Selective isolation of trypsin inhibitor and lectin from soybean whey by chitosan/tripolyphosphate/genipin co-crosslinked beads. Int. J. Mol. Sci..

[17] Medellín-Castillo NA (2021). Formaldehyde and tripolyphosphate crosslinked chitosan hydrogels: Synthesis, characterization and modeling. Int. J. Biol. Macromol..

[18] Ibrahim MNM, Balakrishnan RS, Shamsudeen S, Bahwani SA, Adam F (2012). A concise review of the natural existance, synthesis, properties, and applications of syringaldehyde. BioResources.

[19] Boca SC (2011). Uptake and biological effects of chitosan-capped gold nanoparticles on Chinese Hamster Ovary cells. Mater. Sci. Eng. C.

[20] Martínez-Torres AC (2018). Chitosan gold nanoparticles induce cell death in HeLa and MCF-7 cells through reactive oxygen species production. Int. J. Nanomed..

[21] Kavianinia I, Plieger PG, Kandile NG, Harding DR (2014). In vitro evaluation of spray-dried chitosan microspheres crosslinked with pyromellitic dianhydride for oral colon-specific delivery of protein drugs. J. Appl. Polym. Sci..

[22] Kasaai MR, Arul J, Charlet G (2000). Intrinsic viscosity–molecular weight relationship for chitosan. J. Polym. Sci. Part B Polym. Phys..

[23] Gomha S, Riyadh SM, Mahmmoud EA, Elaasser MM (2015). Synthesis and anticancer activities of thiazoles, 1, 3-thiazines, and thiazolidine using chitosan-grafted-poly (vinylpyridine) as basic catalyst. Heterocycles.

[24] Mosmann T (1983). Rapid colorimetric assay for cellular growth and survival: Application to proliferation and cytotoxicity assays. J. Immunol. Methods..

[25] Abo-Ashour MF (2019). 3-Hydrazinoisatin-based benzenesulfonamides as novel carbonic anhydrase inhibitors endowed with anticancer activity: Synthesis, in vitro biological evaluation and in silico insights. Eur. J. Med. Chem..

[26] Aubry JP (1999). Annexin V used for measuring apoptosis in the early events of cellular cytotoxicity. Cytom. J. Int. Soc. Anal. Cytol..

[27] Kavianinia I, Plieger PG, Kandile NG, Harding DR (2012). New hydrogels based on symmetrical aromatic anhydrides: Synthesis, characterization and metal ion adsorption evaluation. Carbohydr. Polym..

[28] Ahmed ME, Mohamed HM, Mohamed MI, Kandile NG (2020). Sustainable antimicrobial modified chitosan and its nanoparticles hydrogels: Synthesis and characterization. Int. J. Biol. Macromol..

[29] Liu C (2019). Enhanced oral insulin delivery via surface hydrophilic modification of chitosan copolymer based self-assembly polyelectrolyte nanocomplex. Int. J. Pharm..

[30] Bratskaya S (2019). Chitosan gels and cryogels cross-linked with diglycidyl ethers of ethylene glycol and polyethylene glycol in acidic media. Biomacromolecules.

[31] Tripodo G (2018). Hydrogels for biomedical applications from glycol chitosan and PEG diglycidyl ether exhibit pro-angiogenic and antibacterial activity. Carbohydr. Polym..

[32] Vinodhini PA (2017). FTIR, XRD and DSC studies of nanochitosan, cellulose acetate and polyethylene glycol blend ultrafiltration membranes. Int. J. Biol. Macromol..

[33] Nitsae M, Madjid A, Hakim L, Sabarudin A (2016). Preparation of chitosan beads using tripolyphosphate and ethylene glycol diglycidyl ether as crosslinker for Cr (VI) adsorption. Chem. Chem. Technol..

[34] Mirza Ali Mofazzal J (2014). Curcumin-loaded chitosan tripolyphosphate nanoparticles as a safe, natural and effective antibiotic inhibits the infection of *Staphylococcus aureus* and *Pseudomonas aeruginosa* in vivo. Iran J. Biotechnol..

[35] Parize AL, Stulzer HK, Laranjeira MCM, Brighente IMDC, Souza TCRD (2012). Evaluation of chitosan microparticles containing curcumin and crosslinked with sodium tripolyphosphate produced by spray drying. Química Nova..

[36] Abouelnaga AM, Mansour A, Abou Hammad AB, El Nahrawy AM (2024). Optimizing magnetic, dielectric, and antimicrobial performance in chitosan-PEG-Fe2O3@ NiO nanomagnetic composites. Int. J. Biol. Macromol..

[37] El Hamdaoui L, El Marouani M, El Bouchti M, Kifani-Sahban F, El Moussaouiti M (2021). Thermal stability, kinetic degradation and lifetime prediction of chitosan schiff bases derived from aromatic aldehydes. Chem. Sel..

[38] Chuc-Gamboa MG (2021). Antibacterial behavior of chitosan-sodium hyaluronate-PEGDE crosslinked films. Appl. Sci..

[39] Li K, Zhu J, Guan G, Wu H (2019). Preparation of chitosan-sodium alginate films through layer-by-layer assembly and ferulic acid crosslinking: Film properties, characterization, and formation mechanism. Int. J. Biol. Macromol..

[40] Kaur I, Goyal D, Agnihotri S (2021). Formulation of cartap hydrochloride crosslinked chitosan tripolyphosphate nanospheres and its characterization. Colloid Polym. Sci..

[41] Kandile NG (2023). New sustainable antimicrobial chitosan hydrogels based on sulfonamides and its nanocomposites: Fabrication and characterization. Int. J. Biol. Macromol..

[42] Miguel SP, Moreira AF, Correia IJ (2019). Chitosan based-asymmetric membranes for wound healing: A review. Int. J. Biol. Macromol..

[43] Hamidi M, Azadi A, Rafiei P (2008). Hydrogel nanoparticles in drug delivery. Adv. Drug Deliv. Rev..

[44] Wilson B, Lavanya Y, Priyadarshini S, Ramasamy M, Jenita JL (2014). Albumin nanoparticles for the delivery of gabapentin: preparation, characterization and pharmacodynamic studies. Int. J. Pharm..

[45] Ilyas RA (2022). Natural-fiber-reinforced chitosan, chitosan blends and their nanocomposites for various advanced applications. Polymers.

[46] Neto CDT (2005). Thermal analysis of chitosan based networks. Carbohydr. Polym..

[47] Hamed AA, Abdelhamid IA, Saad GR, Elkady NA, Elsabee MZ (2020). Synthesis, characterization and antimicrobial activity of a novel chitosan Schiff bases based on heterocyclic moieties. Int. J. Biol. Macromol..

[48] Kandile NG, Mohamed HM, Nasr AS (2020). Novel hydrazinocurcumin derivative loaded chitosan, ZnO, and Au nanoparticles formulations for drug release and cell cytotoxicity. Int. J. Biol. Macromol..

[49] Tahari N (2022). Preparation of chitosan/tannin and montmorillonite films as adsorbents for Methyl Orange dye removal. Int. J. Biol. Macromol..

[50] Kittur F, Prashanth KH, Sankar KU, Tharanathan R (2002). Characterization of chitin, chitosan and their carboxymethyl derivatives by differential scanning calorimetry. Carbohydr. Polym..

[51] Kandile NG, Nasr AS (2011). New polyamides and polyesteramides incorporated with bis (carboxy-substituted) hydrazines: Synthesis and characterization. J. Appl. Polym. Sci..

[52] Kandile NG, Nasr AS (2011). Hydrogels based on a three component system with potential for leaching metals. Carbohydr. Polym..

[53] Saeedi F, Montazeri A, Bahari Y, Pishvaee M, Ranjbar M (2018). Synthesis and characterization of chitosan-poly vinyl alcohol-graphene oxide nanocomposites. Int. J. Chemoinform. Chem. Eng..

[54] Šimat V (2020). Recent advances in marine-based nutraceuticals and their health benefits. Mar. Drugs.

[55] Chen H (2021). Zinc oxide nanoparticles synthesized from *Aspergillus terreus* induces oxidative stress-mediated apoptosis through modulating apoptotic proteins in human cervical cancer HeLa cells. J. Pharm. Pharmacol..

[56] Grobler S (2014). Cytotoxic effect of chitosan-H, resveratrol, β-Carotene and propolisand their chitosan hydro-gels on Balb/C mouse 3T3 fibroblast cells. Int. J. Dent. Oral Sci..

[57] De Matteis L (2016). Controlling properties and cytotoxicity of chitosan nanocapsules by chemical grafting. Mar. Drugs.

[58] Ambrosone A, Matteis LD, Serrano-Sevilla I, Tortiglione C, De La Fuente JM (2020). Glycogen synthase kinase 3β inhibitor delivered by chitosan nanocapsules promotes safe, fast, and efficient activation of Wnt signaling in vivo. ACS Biomater. Sci. Eng..

[59] Kandile NG, Ahmed ME, Mohamed MI, Mohamed HM (2024). Therapeutic applications of sustainable new chitosan derivatives and its nanocomposites: Fabrication and characterization. Int. J. Biol. Macromol..

[60] Lee S-H, Ryu B, Je J-Y, Kim S-K (2011). Diethylaminoethyl chitosan induces apoptosis in HeLa cells via activation of caspase-3 and p53 expression. Carbohydr. Polym..

[61] Danial NN, Korsmeyer SJ (2004). Cell death: Critical control points. Cell.

[62] Abd Elgadir M (2015). Impact of chitosan composites and chitosan nanoparticle composites on various drug delivery systems: A review. J. Food Drug Anal..

[63] Baksi R (2018). In vitro and in vivo anticancer efficacy potential of Quercetin loaded polymeric nanoparticles. Biomed. Pharmacother..

[64] Li J, Zhuang S (2020). Antibacterial activity of chitosan and its derivatives and their interaction mechanism with bacteria: Current state and perspectives. Eur. Polym. J..

[65] Barrera-Martínez CL (2021). Chitosan microparticles as entrapment system for trans-cinnamaldehyde: Synthesis, drug loading, and in vitro cytotoxicity evaluation. Int. J. Biol. Macromol..

[66] Babu A, Ramesh R (2017). Multifaceted applications of chitosan in cancer drug delivery and therapy. Mar. Drugs.

[67] Naskar S, Sharma S, Kuotsu K (2019). Chitosan-based nanoparticles: An overview of biomedical applications and its preparation. J. Drug Deliv. Sci. Technol..

[68] Yu X (2015). Intracellular targeted co-delivery of shMDR1 and gefitinib with chitosan nanoparticles for overcoming multidrug resistance. Int. J. Nanomed..

[69] Sanni L (2022). Statistical methods for food science: Introductory procedures for the food practitioner. Int. J. Food Sci. Technol..

[70] Özdamar, B., Sürmeli, Y. & Mohamed, G. Ş. Cytotoxic and apoptotic effects of olive leaf extract chitosan nanoparticles on breast cancer MCF-7 and lung cancer A549 cell. 1–16. 10.21203/rs.3.rs-2209453/v1 (2022).

[71] Parida UK, Rout N, Bindhani BK (2013). In vitro properties of chitosan nanoparticles induce apoptosis in human lymphoma SUDHL-4 cell line. Adv. Biosci. Biotechnol..

